# Goat Milk Fat Naturally Enriched with Conjugated Linoleic Acid Increased Lipoproteins and Reduced Triacylglycerol in Rats

**DOI:** 10.3390/molecules19033820

**Published:** 2014-03-24

**Authors:** Raphaela Rodrigues, Juliana Soares, Hugo Garcia, Claudenice Nascimento, Maria Medeiros, Marco Bomfim, Maria Carmo Medeiros, Rita Queiroga

**Affiliations:** 1 Departament of Nutrition, Universidade Federal de Campina Grande, Cuité 58175-000, PB, Brazil; 2 Departament of Morfology, Universidade Federal da Paraíba, João Pessoa 58051-900, PB, Brazil; 3 Escola Técnica de Saúde, Universidade Federal da Paraíba, João Pessoa 58051-900, PB, Brazil; 4 Departament of Physiology and Pathology, Universidade Federal da Paraíba, João Pessoa 58051-900, PB, Brazil; 5 Empresa Brasileira de Pesquisa Agropecuária, EMBRAPA Caprinos e Ovinos, Sobral 62010-970, CE, Brazil; 6 Departament of Nutrition, Universidade Federal de Pernambuco, Recife 50670-901, PE, Brazil; 7 Departament of Nutrition, Universidade Federal da Paraíba, João Pessoa 58051-900, PB, Brazil

**Keywords:** lipids, goat milk, steatosis, cholesterol, lipoprotein, rats

## Abstract

Goat milk is source of different lipids, including conjugated linoleic acid (CLA). CLA reduces body fat and protect against cardiovascular diseases. In the present study fat from goat milk naturally enriched with CLA was used. Male Wistar rats were divided into three groups that received during a 10 week diet with different lipid sources: soybean oil (CON), coconut oil (CO) and goat milk fat naturally enriched with CLA (GM-CLA). We evaluated the effects of a GM-CLA on biochemistry parameters - high density lipoprotein (HDL), triacylglycerol (TAG), TAG/HDL ratio, total cholesterol and glucose -, body weight and histopathological aspects of the intestine and liver. GM-CLA increased body weight from the second to the fifth week of the experiment compared to CON. Feed intake differed between the CON group and GM-CLA early in the first to third week of the experiments and later between the ninth and tenth week. The CLA-diet group showed increased levels of HDL, reduced levels of TAG and TAG/HDL ratio and no effect on LDL, but enhanced total cholesterol. Serum glucose of the GM-CLA group showed no difference from the control group. Thus, a GM-CLA diet promoted growth in young rats and acted as protector of cardiovascular function, but further studies are still needed to clarify these effects.

## 1. Introduction

Nowadays, one of the main causes of death worldwide is cardiovascular disease (CVD) [1]. In this context, nutrition has an important role, because of its ability to modulate different risk factors for CVD, such as overweight and obesity, dyslipidemia, atherosclerosis, hypertension, insulin resistance and glucose intolerance [2]. Among the components of the diet, lipids are an important modulator of metabolism. Each specific lipid type has different effects on the serum lipid profile and body composition. In general, saturated fatty acids (SFA) are hypercholesterolemic, while polyunsaturated fatty acids (PUFA) and monounsaturated fatty acids (MUFA) are hypocholesterolemic [3,4]. Among the fatty acids that can modulate lipid metabolism, is conjugated linoleic acid (CLA).

CLA is a term used to designate a set of positional and geometric isomers of octadecadienoic acid, which has two conjugated double bonds. The most common forms are *9-cis,11-trans-* and *10-trans,12-cis-*. CLA was first described in a study by Pariza and Hargraves [5] as a compound present in roast beef with anti-mutagenic action. Since then, several studies had attributed to CLA diverse biological activities such as reduction of body weight and body fat [6,7,8,9], lean mass increase, improvement of the serum lipid profile [6], cardiovascular function protection [6,10], and anticarcinogenic action [11,12]. Conjugated linoleic acid is naturally produced by biohydrogenation of linoleic acid by bacteria in the rumen of animals, thus the main sources of this fatty acid for humans are dairy and meat products from ruminants, such as goat milk.

Goat milk is a food of high nutritional value, with high biological value protein, and a good source of short-chain fatty acids and medium chain fatty acids, minerals and vitamins [13]. The fat from this product has better digestibility, the protein has smaller allergenic potential and this milk also has less lactose than cow milk [13]. Furthermore, goat milk provides a better use of iron, which minimizes possible interactions between iron and other minerals such as calcium, phosphorus and magnesium [14,15,16]. In consequence of the nutritional importance of goat milk and the emergent interest in CLA, different studies have been undertaken in order to increase the amount of this compound in goat milk by manipulation of the animal feed composition, especially the fat [17,18]. However, fat from goat milk is not a good source of n-3 and n-6, but when goats are fed with unsaturated fatty acids the concentration of CLA in their milk increases [18]. Preliminary research used a goat milk lipid naturally enriched with CLA [19]. To obtain these lipids, goats were feed with soybean oil, naturally increasing CLA in the milk. After this when goat milk fat naturally enriched with CLA was offered to rats during pregnancy and lactation, was observed that it altered body weight and brain development in the offspring [19]. However, the effects of this diet in adult animals remained unknown.

Based on these finding, we hypothesized that the lipids from goat milk naturally enriched with CLA can affect the metabolism of nutrients, reflecting on the physical, biochemical and histological findings. The present study aimed to evaluate the effects of a diet with goat milk fat naturally enriched with CLA on lipid profile, body weight and histopathological aspects of the intestine and liver of rats, compared with diets composed of different lipid sources.

## 2. Results and Discussion

### 2.1. Body Weight and Feed Intake

**Figure 1:**
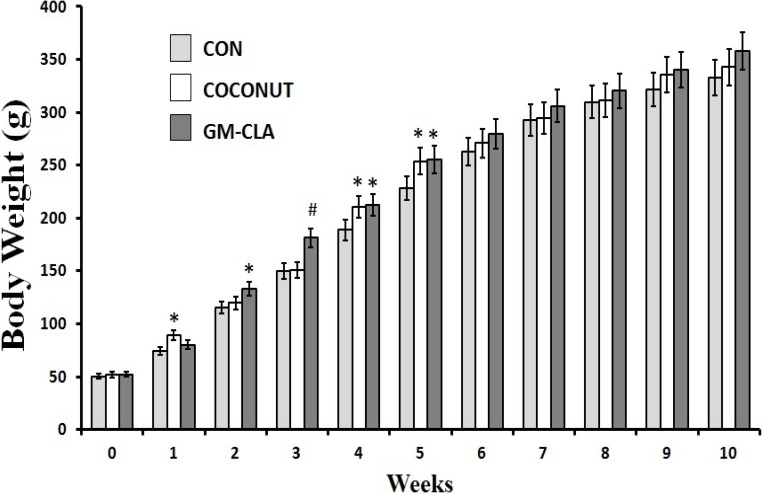
Body weight (g) for each week of experimental treatment (means ± sd). The number of animals in the groups is 12. An (*) denotes a significant difference (*p* ≤ 0.05) from control group. An (^#^) denotes a significant difference (*p* ≤ 0.05) from control and coconut groups.

**Figure 2:**
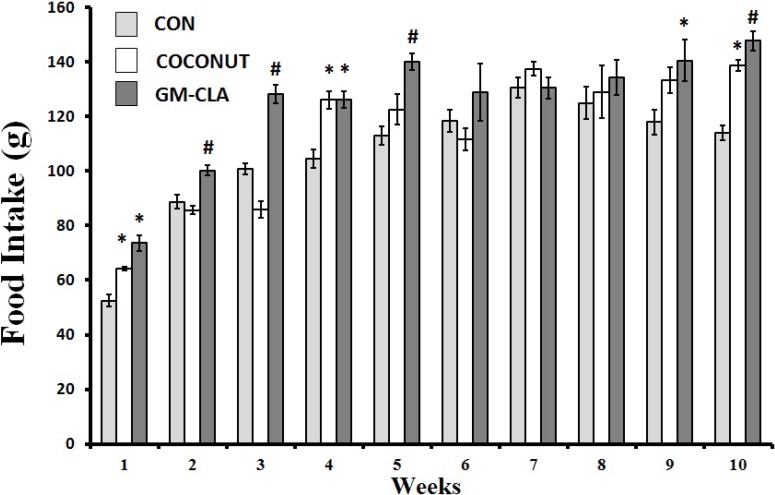
Weekly means of food intake for each experimental group. The number of animals in the groups is 12. An (*) denotes a significant difference (*p* ≤ 0.05) from control group. An (^#^) denotes a significant difference (*p* ≤ 0.05) from control and coconut groups.

Body weight at the end of treatment did not differ between the CON, CO and GM-CLA groups (Figure 1), with means of 332.91 g, 342.72 g and 357.70 g, respectively. The means for body weight were higher (*p* < 0.05) in the GM-CLA group at weeks 2, 3, 4 and 5 when compared with the CON group. The CO group at weeks 4 and 5 showed a increased body weight in comparison with the CON group. This group had higher body weight (*p* < 0.05) than the CON group for weeks 1, 4 and 5. The CO and GM-CLA groups showed a trend to a higher consumption of feed as demonstrated in Figure 2. The mean of total feed intake for each animal of the CO group (1,171.55 g ± 64.85) was greater than in the CON group (1,064.69 g ± 55.88) (*p* < 0.05). The GM-CLA total feed intake (1,250.98 g ± 71.90) was significantly higher than that of the CO and CON groups (*p* < 0.05).

From the second to the fifth week of experiments, the body weight of the GM-CLA group was higher than the CON one, but this difference disappeared at the end of the period of ten weeks. A similar result was found by a study with rats given a diet of meat containing CLA in a manner similar to goat milk used in this study, where a greater body weight was observed in the initial weeks that eventually disappeared during the experiment [20]. The highest body weights in the initial weeks should be emphasized, since it corresponds to the initial phase of animal growth, suggesting a growth-promoting effect by GM-CLA. Other studies have shown the effect of CLA as a growth factor for young rats, conveyed as a greater efficiency of food assimilation and increased body weight gains [21,22]. On the other hand, when isolated CLA is used, this gain does not occur [23], thus the importance of the presence of other fatty acids derived from goat milk, which may be more favorable to growth and consequent body mass gain of the animals, should be considered. The lack of difference between the weight of the GM-CLA group compared to other groups, found at the end of the trial period, were consistent with studies with CLA on animal models [24,25]. In studies comparing the effect of different isomers of CLA on weight and body composition of rats, it was found that the 9-*cis*, 11*-trans*- isomer did not significantly alter the body weight in relation to the control group [26,27]. This fact is interesting for our study because this is the predominant form found naturally in foods, like the CLA used in our study. However, the food intake was higher in the GM-CLA group in the initial weeks of the experiment. This could explain an increased body weight gain. Animal foods such as milk and meat [20], can show better palatability resulting in higher food consumption as demonstrated in the GM-CLA group. The CLA by itself, does not seem to affect the feed intake [7,9], or even decrease the total feed intake [28], so it is suggested that the fat in goat milk would be responsible for better acceptance of this food by animals.

### 2.2. Biochemical Analysis

The values of TC, 67.17 (±7.60) mg/dL and 73.83 (±5.00) mg/dL, HDL, 16.08 (±1.86) mg/dL and 15.22 (±1.58) mg/dL, and glucose, 82 mg/dL and 65 mg/dL did not differ between CON and CO (*p* > 0.05), respectively. The levels of TAG were significantly higher in group CO, 64 (±4.12) mg/dL, and lower in group GM-CLA, 35.5 (±3.66) mg/dL. The GM-CLA group showed the highest values of TC (*p* < 0.001) and HDL (*p* < 0.001), 87.00 (±11.38) mg/dL and 26.63 (±3.16) mg/dL, respectively, compared with the other groups however had a lower TAG/HDL ratio Table 1. There were no differences on LDL between the three groups. The serum glucose GM-CLA was higher when compared to CO (*p* < 0.05) but did not differ from CON.

**Table 1:** Means values for total cholesterol (CT), HDL-cholesterol (HDL), triglycerides (TG), TG/HDL ratio and glucose from animals treated with different lipids source during ten weeks.

Parameter	Control	Coconut Oil	Goat Milk Fat
**Total Cholesterol**	67.17 (±7.60)	73.83 (±5.00)	87.00 (±11.38) ^#^
**HDL**	16.08 (±1.86)	15.22 (±1.58)	26.63 (±3.16) ^#^
**LDL**	50.01 (±18.08)	42.81 (±8.45)	53.27 ±8.59)
**TAG**	40.78 (±4.84)	64.00 (±4.12) ^*^	35.50(±3.66) ^#^
**TG/HDL**	2.82 (±1.22)	4.06 (±0.71) ^*^	1.36(±0.26) ^#^
**Glucose**	81.33(±7.94)	64.86(±4.56)	107.88(± 8.5) §

Values are means (± SD). The diets are similar except for the fat source: soybean oil, coconut oil and goat milk fat source of CLA. The number of animals in each group is 8. Means (±SD) that doesn’t share the same letter differ significantly. ^#^ *p* < 0.05 *vs.* control and coconut group; ^*^ *p* < 0.05 *vs.* control group; § *p* < 0.05 *vs.* coconut group.

Dietary fat generally is able to influence the serum lipid profile of animals involved in the metabolism of lipids in the body of the animal, determining the synthesis or removal of blood lipids. In this study, we evaluated the total cholesterol and HDL and LDL, TAG, and the TAG/HDL ratio, which are identified as important risk factors for CVD. Biochemistry analysis of blood samples from the animal showed that the levels of TAG presented by the GM-CLA group were in agreement with results found in a study with goat’s milk that decreased TAG [29]. Additionally, serum TAG can be reduced by CLA [6,30] acting on lipogenesis (by suppression of SREBP-1c) or by increased fatty acid oxidation-induced suppression of PPAR gamma, which reduces the pool of substrate for the production of TAG [31,32]. With regard to HDL, the GM-CLA group had the largest recorded serum levels, as shown in other studies with supplementation by CLA [33,34]. Moreover, the reduction of TAG and increased HDL observed in GM-CLA group lead to reduced values of the TAG/HDL ratio. Similar results can be observed in other studies with animal models that received CLA [35]. Beneficial alterations demonstrated by GM-CLA, strengthens the hypothesis that CLA may be a protective dietary factor for CVD. However, we observed an increase of total cholesterol, consistent with a study that used isolated isomers of CLA [36], this alteration could be related to the capacity of CLA to increase the body fatty mobilization [37].

We observed a significant difference in serum glucose between the GM-CLA and CO groups, but not between the CON and GM-CLA groups, although there was a trend to higher values, similar to research using soybean oil as a control group [7]. The development of insulin resistance, with consequent increase in blood glucose has been reported in studies with administration of CLA [9,37]. These outcomes could be explained by hypotheses as a deviation of the substrate used for the production of energy from carbohydrates to lipids, and decreased insulin signaling to activate inflammatory pathways and stress kinases. The serum glucose observed in the GM-CLA was consistent with a study of the biochemical parameters of Wistar rats from a bioterium of Universidade Federal da Paraiba [38].

### 2.3. Histopathological Analysis

**Figure 3:**
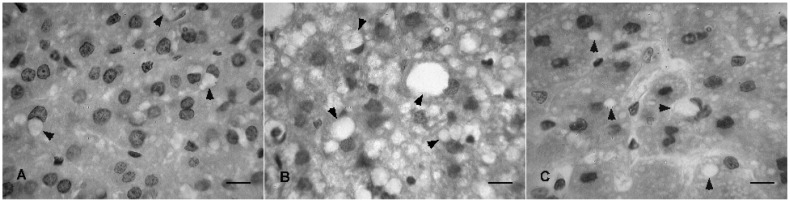
Histological sections of rat’s liver from the experimental groups: GM-CLA (**A**) CO (**B**) and CON (**C**). Arrows indicates fat accumulations on liver cells, characteristic of hepatic steatosis. Scale bar = 10 μm.

We observed the presence of intracellular fat globules in the liver in all experimental groups, which rangec from mild to moderate in animals of the CON and GM-CLA groups, and moderate to severe in the CO group (Figure 3). In the intestine no pathological changes were observed (data not shown).

The occurrence of fat liver is consistent with other studies using animals which have been administered CLA [39,40]. One possible way by which CLA cause hepatic steatosis would be a transfer of fatty acids derived from adipose tissue into the hepatocytes, and action on genes regulating liver lipid metabolism [39,41]. Furthermore, the marked reduction of serum TG may be related to such a high accumulation of fat in the liver [42]. However, other studies showed the opposite effect, with inhibition of hepatic fat deposition and preservation of the liver histological characteristics [2,43]. As the deposition of fat in the liver was observed in all groups, such a modification should not be attributed to the consumption of dietary containing CLA. Moreover, considering that the animals consumed an isocaloric diet that differed only in the quality of fat, these alterations could be induced by the utilization of purified diet [44]. The maintenance of the histological characteristics of the intestine shows a safety for the administration of GM-CLA on this organ.

## 3. Experimental

### 3.1. Obtainment of the Lipids from Goat Milk

To increase CLA in goat’s fat milk, 3.5% of soybean oil was added in the goat feed. The adaptation period was twenty days and when this period ended, the milk was collected after five days. After collection, the milk was skimmed, frozen and stored to be used in the rat’s diet. We followed the general procedures described by Stanciuc *et al.* [45]. The fatty acid composition of the fat from goat milk is presented in Table 2.

**Table 2:** Fatty acid profile of the different fat sources (%).

Fatty acids	Control	Coconut Oil	Goat Milk Fat
C4:0	-	-	-
C6:0	-	-	0.96
C8:0	-	-	0.99
C10:0	-	-	3.60
C12:0	-	47.92	2.10
C14:0	0.42	14.83	6.40
C15:0	-	-	0.63
C16:0	17.1	9.34	24.26
C16:1	-	-	0.34
C17:0	0.34	-	0.53
C18:0	5.93	4.25	20.94
C18:1(n9)	28.53	19.2	28.79
C18:2(n6)	42.56	4.45	5.97
C18:2 *CLA	-	-	1.20
C18:3(n3)	4.89	-	0.63
Not Determined	0.23	0.01	2.66

* CLA = Conjugated linoleic acid.

### 3.2. Animals and Diet

Male Wistar rats (21 days of age) were obtained from the Department of Nutrition of Universidade de Pernambuco, Brazil and housed in individual cages under standard temperature (22 ± 1 °C) and humidity (65% ± 5%) and a light/dark cycle of 12 h/12 h (artificial lights, 6:00 a.m. to 6:00 p.m.). The rats were then randomly divided into three groups (with 12 animals each) and fed for 10 weeks with experimental diets formulated with different lipid sources in accordance with the group allocated: control group (CON), receiving soybean oil; coconut (CO), receiving coconut oil; and goat milk (GM-CLA), receiving goat milk fat (as a source of CLA). Diets were formulated based on recommendations of the American Institute of Nutrition (AIN-93) [46] and manufactured by the company Rhoster (Sao Paulo, Brazil), being stored under refrigeration. All procedures were previously approved by the Ethics Committee on Animal Research of the Pharmaceutical Technology Laboratory, Universidade Federal da Paraiba. The composition of the diet (%) consisted of 19% casein, 54% cornstarch, 10% sucrose, 7% fat, 5% cellulose, 3.5% mineral mix, 0.25% choline, 0.3% D, L-cystine and 1% vitamin mix (AIN-93). During all the experiment, the rats received food and water *ad libitum*. Body weight and food consumption were recorded weekly.

### 3.3. Measurement of Dietary Fatty Acids

The extraction of fatty acids from goat’s milk was performed by the use of a methanol-chloroform technique, following the methodology proposed by Bligh and Dyer [47]. Fatty acids were identified by comparison of retention times and the percentages of fatty acids were obtained through the software—GC Solution (Shimadzu Corporation, Kyoto, Japan), with the authentic standard fatty acids from Sigma-Aldrich (St. Louis, MO, USA), FAME 37 and conjugated linoleic acid mixture (CLA). Fatty acids were quantified by normalization of methyl ester areas and the results were expressed as percentage of total fatty acids (%). Fatty acid methyl esters were analyzed by gas chromatography, using a GC-FID (HP-6890-Agillent), equipped with a flame ionization detector, capillary column (SP-2560, Supelco, Bellefonte, PA, USA), stationary phase fused silica with 100 m long, 0.25 mm internal diameter 0.20 mm film thickness. We used nitrogen as carrier gas at a flow rate of 1.8 mL/min, nitrogen (50 mL/min) and hydrogen (400 mL/min) as detector and synthetic air (400 mL/min). The oven temperature program was initial 50 °C, with standby time of 3 min, rising to 150 °C (4 °C/min) and remaining at this temperature for 1 min. Then, at a rate of 1 °C/min, the temperature was raised to 170 °C and maintained for 1 min. Subsequently, the temperature rose to 220 °C (8 °C/min) and held for 30 min. The whole process amounted to 86.25 min. The injector temperature was 250 °C and the detector was 300 °C. The fatty acids were identified by comparing the retention times of the methyl esters of the samples with the patterns: BCR-CRM 164 Anhydrous Milk-Fat Producer (BCR Institute for Reference Materials and Measurements (certified butter) and Supelco TM Component FAME Mix, cat 18919 (a mixture of 37 isolated FAs).

### 3.4. Biochemical Analysis

At the end of the experiment, animals were anesthetized with ketamine and xylazine (1 mL/kg), subjected to cardiac puncture for the withdrawal of blood samples. The biochemical measurements of glucose, total cholesterol (TC), triacylglycerol (TAG) proceeded by the enzymatic Trinder method (Labtest-Diagnosis, St. Louis, MO, USA). To determine the high-density lipoprotein cholesterol (HDL) used the methodological principle employed by Rautela and Liedtek [48]. Very-low-density lipoprotein cholesterol (VLDL) was calculated as triacylglycerol/5. The low-density lipoprotein cholesterol (LDL) was estimated by the difference between total cholesterol and (VLDL+HDL) [49]. To these determinations the animals was randomized and only eight animals were used.

### 3.5. Tissue Preparation

Liver and intestine were immediately removed after the sacrifice and fixed in Bouin’s solution for 48 h, dehydrated, infiltrates and embedded in paraffin wax, sectioned at 5 µm on rotary microtome Leica RM 2125 RT model. Sections were stained with hematoxylin and eosin. Images were taken with a photomicroscope and histopathological analysis.

### 3.6. Statistical Analysis

Body weight, feed intake and biochemical parameters were expressed as mean values. These data were subjected to analysis of variance to compare three independent samples. The difference between the means was defined by the Tukey test and statistical significance was set at 5% (*p* < 0.05).

## 4. Conclusions

In conclusion, our data documented for the first time the effect of a goat milk lipids naturally enriched with CLA on: (1) biochemical and (2) histological (not causing steatosis) parameters in rats. The lipids from goat milk naturally enriched with CLA induced a low risk of cardiovascular disease. These findings are important given that cardiovascular diseases are associated with metabolic syndrome. This experimental diet is thus worthy or further consideration as a functional food demonstrating a potential cardiac function protective effect.
